# Growth Hormone Promotes *in vitro* Maturation of Human Oocytes

**DOI:** 10.3389/fendo.2019.00485

**Published:** 2019-07-24

**Authors:** Yue Li, Hui Liu, Qingqing Yu, Hongbin Liu, Tao Huang, Shigang Zhao, Jinlong Ma, Han Zhao

**Affiliations:** ^1^Center for Reproductive Medicine, Shandong University, Jinan, China; ^2^The Key Laboratory of Reproductive Endocrinology of Ministry of Education, Jinan, China; ^3^National Research Center for Assisted Reproductive Technology and Reproductive Genetics, Jinan, China

**Keywords:** growth hormone, human oocyte, *in vitro* maturation, single-cell RNA-seq, *AURKA*, *PDIA6*, *LINGO2*, *CENPJ*

## Abstract

Increasing the success rate of *in vitro* maturation (IVM) for human oocytes has a major clinical significance. Previous studies have shown that growth hormone (GH) added into IVM medium could promote IVM of oocytes from non-human beings. However, few studies on systematic IVM for human oocytes with GH have been reported. Human germinal vesicle (GV) oocytes collected for IVM were cultured with different concentrations of GH to optimize the concentration. Metaphase II (MII) stage oocytes obtained from IVM were fertilized by intracytoplasmic sperm injection (ICSI). Maturation rate, fertilization rate, and blastocyst rate were assessed after IVM with or without GH. Furthermore, gene expression profiles were compared in oocytes between the two groups using single-cell RNA-seq. The optimal concentration of GH for IVM was 200 ng/ml, and the maturation rate of this group reached 70% which was double that of the control group (35%, *P* = 0.004). The fertilization rate (73.1 vs. 60.3%) and blastocyst rate (25.0 vs. 15.5%) both had an increasing trend in the GH group compared to controls. Single-cell RNA-Seq and real-time PCR data showed that GH could significantly enhance the expression of genes associated with meiotic progression and embryo development, such as *AURKA* (aurora kinase A, *P* = 0.007), *PDIA6* (protein disulfide isomerase family A member 6, *P* = 0.007), *LINGO2* (leucine rich repeat and Ig domain containing 2, *P* = 0.007), and *CENPJ* (centromere protein J, *P* = 0.039). Taken together, GH could promote maturation of human oocytes, probably through accelerating meiotic progression, balancing redox homeostasis of cellular environment, and promoting oocyte developmental competence.

## Introduction

Oocyte *in vitro* maturation (IVM) is a patient-friendly and less expensive human assisted reproduction technology (ART). It can effectively decrease the risk of ovarian hyperstimulation syndrome (OHSS) due to its low dosage of gonadotropin administration, and can also simplify treatment procedures by avoiding frequent ultrasonography. This technology can be applied to patients of polycystic ovary syndrome (PCOS) who have high risk of OHSS, patients with gonadotrophin resistance, and patients who have contraindications for ovulation stimulants. In addition, IVM can be used in fertility preservation. However, owing to the low success rate and reduced oocyte developmental potentiality compared to conventional *in vitro* fertilization (IVF), IVM is difficult to be widely adopted in clinical practice. Thus, improving the success rate by optimizing the IVM culture system has a major clinical significance.

Growth hormone (GH) as a classical and pleiotropic peptide hormone has been paid more attention for its administration in ovarian co-stimulation for infertile women. Among distinct groups of infertile patients, GH applied *in vivo* has been reported to make effects on assisted reproduction outcomes. For the patients who responded normally, the treatment of GH could raise the implantation rate and pregnancy rate (1). For poor ovarian responders, women of advanced age, and patients with multiple IVF failures, GH treatment could also improve the outcome of assisted conception by increasing the number of metaphase II (MII) stage oocytes, two-pronuclear zygotes (2PN) and transferred embryos (2–6). *In vivo* administration of GH has also been reported to promote IVM of human germinal vesicle (GV) stage oocytes (7).

In addition to application *in vivo*, the positive effects of GH added into IVM medium have been intensively investigated in many species of animals. Related studies showed that GH could promote oocytes maturation and embryo development in bovine (8–14), ovine (15), equine (16–19), porcine (16), rat (20), mouse (21), canine (22), and rhesus macaque (23). Its facilitatory role in IVM and embryo development of animals can enlighten us on the advancements of human IVM.

Few studies on systematic IVM for human oocytes with GH have been reported. To examine the effects of GH on human oocyte IVM, the immature oocytes derived from ICSI cycles could be made use of to perform a preliminary study. These oocytes are often discarded and considered to be useless. However, these immature oocytes have their own value. They can achieve maturation and early embryo development *in vitro* (24), and obtain pregnancy (25). If these oocytes could be rescued to maturation for clinical purposes, the patients may benefit from it. There has been a case report showing a successful pregnancy and delivery through one naked GV oocyte from stimulated ovary cultured to maturation with GH *in vitro* (26).

In this study, we gathered GV-stage oocytes from ICSI patients to identify whether GH works during human oocyte IVM and to clarify the optimal GH concentration. Then single-cell RNA-seq was employed to explore the mechanism of GH.

## Materials and Methods

### Participants

The GV-stage oocytes were donated by patients who underwent ICSI treatment due to male factors at the Center for Reproductive Medicine, Shandong University.

### Ethical Approval

The study was approved by the Institutional Review Board of Reproductive Medicine, Shandong University. Each participant in the study had written the informed consent. The blastocysts in this study, which were formed by both oocytes and sperms donated for research, would not be used for any reproductive purpose and were all destroyed after observation.

### Oocytes Collection

Follicles were punctured under ultrasound guidance 36 h after the administration of 10,000 IU human chorionic gonadotropin (hCG, Merck Serono, Switzerland). The corona-cumulus cells were removed by hyaluronidase (Irvine Scientific, USA), and then the meiotic status of human oocytes was assessed. Only MII-stage oocytes were fertilized by ICSI for the patients. Those GV-stage oocytes with a discernable germinal vesicle were donated and collected for this study.

### IVM and ICSI

The GV-stage oocytes obtained in each experimental day were randomly distributed to different concentration groups including one control group, and we always cultured fresh GV oocytes. An accumulating total of 252 GV-stage oocytes were made use of and divided into eight groups with different concentrations of GH. The concentration gradients were set as 0 (control), 10, 50, 100, 200, 300, 500, 1,000ng/ml (8, 16, 27). They were cultured for 24 h in the IVM medium, which was Medium 199 (Gibco/life technologies, USA) and meanwhile supplemented with 0.29 mmol/L sodium pyruvate (Sigma, USA), 10% human serum albumin (Vitrolife, Sweden), 0.075 IU/mL recombinant follicle stimulating hormone (FSH, Merck Serono, Switzerland), 0.15 IU/mL hCG, and 10 ng/mL epidermal growth factor (EGF, Sigma, USA). Then the number of MII-stage oocytes were counted, and the criterion of nuclear maturation was the extrusion of the first polar body. Then the sperms donated for research were used to perform ICSI for MII-stage oocytes obtained through IVM. ICSI was carried out under an inverted microscope (Nikon, Japan), and all procedures employed sequential culture media supplied by Vitrolife (G-IVF, G1 and G2, Sweden). 16 to 18 hours after ICSI, the conditions of fertilization were observed and the number of two pronuclear zygotes (2PN) was counted. Five to six days after fertilization, the number of blastocysts was determined. All embryos were incubated up to the blastocyst stage according to Gardner's criteria (28). The fertilization rate was the percentage of number of 2PN accounting for number of MII oocytes. The blastocyst rate was the percentage of number of blastocysts accounting for number of MII oocytes.

### Oocyte RNA Sequencing

Three pairs of GV-stage oocytes from three patients respectively were cultured for 24 h with 200 ng/ml GH or not and then washed with phosphate-buffered saline (PBS). Each oocyte was collected and transferred into a 0.2 ml RNase-free microcentrifuge tube containing 2 μl lysis buffer, which was composed of 0.2% Triton X-100 (Sigma, USA) and 2 U/μl of RNase inhibitor (Clontech, USA). Then the cDNA was obtained by the Smart-Seq 2 method (29). For each sample, 20 ng cDNA was used for the Library construction. Then the constructed Library was sequenced on the platform of Illumina HiSeq.

### Sequencing Data Analyses

By using the sequencing strategy of PE150, pair-end reads were obtained. The software of fastqc was used for the quality control analysis of the reads. Using Tophat2, the reads were aligned to reference genome, which was downloaded from the Ensemble database. After that, the software of RseQC was used to do the quality control for the alignment data. According to the reads alignment results, reads were assigned to specific transcripts to count the transcript reads. TPM (Transcripts per Million) was adopted and used for standardization. Reads were aligned to mRNA sequences by the bowtie software and the mRNA quantitative analyses were done using eXpress software. Differential Expression Analysis of mRNA was performed by DESeq software. In the case of biological duplication, differentially expressed mRNA was filtered by *P-*value and adjusted *P*-value, which was the *P-*value after multiple comparison correction.

### Real-Time PCR for Validation

The sequencing results were validated by real-time PCR using nine pairs of oocytes from nine patients, respectively. The cDNA from these 18 oocytes was obtained through REPLI-g WTA Single Cell Kit (Qiagen, Germany) and was used for real-time PCR. Real-time PCR was performed with SYBR Premix Ex Taq (Tli RNaseH Plus) (Takara Bio Inc., Japan) in the LightCycler 480 II (Roche, Germany). Gene specific primers were designed by Primer Premier 5.0 Software (Premier Inc., Canada; Supplementary Table S1). The relative expression level of these genes was normalized by the housekeeping gene of *GAPDH*.

### Statistical Analysis

Categorical data were displayed as counts and percentages and analyzed by chi-square test. Real-time PCR data were counted and analyzed using the method of 2^−ΔCT^ (30), expressed as the mean ± SEM, and compared by paired-samples *t-*test with two tails. Statistical analyses were performed with SPSS software, and statistical significance was considered as *P* < 0.05.

## Results

### Optimal Concentration of GH for Human Oocyte IVM

As shown in Table 1, a total of 252 GV-stage oocytes were cultured in eight groups with different GH concentrations. After IVM, the maturation rate of control group was 35%, while in 200 ng/ml GH group, the maturation rate was the highest (70%, *P* = 0.004).

**Table 1:** Maturation rates of IVM according to different GH concentrations.

**Groups (GH concentration ng/ml)**	**No. of GV oocytes**	**No. of MII oocytes (24 h)**	**Maturation rate (%)**
0	40	14	35.0^a^
10	30	15	50.0
50	31	18	58.1
100	31	17	54.8
200	30	21	70.0^a^
300	30	20	66.7
500	30	17	56.7
1,000	30	15	50.0

a *P < 0.05*.

### GH May Have an Effect on Fertilization and Early Embryo Development

After the optimal GH concentration for IVM was confirmed, MII-stage oocytes (*N* = 52) cultured with 200 ng/ml GH and MII-stage oocytes (*N* = 58) from the control group were collected and fertilized by ICSI. The fertilization rate of the GH group was 73.1%, which was higher than 60.3% of the control group (*P* = 0.158). The blastocyst rate of the GH group was 25.0%, which was higher than 15.5% of the control group (*P* = 0.214). But these two differences were both of no statistical significance (Table 2).

**Table 2:** Fertilization rates and blastocyst rates between GH and the control group.

	**Control**	**GH (200 ng/ml)**	***P*-value**
No. of MII	58	52	
No. of 2PN (fertilization rate)	35 (60.3%)	38 (73.1%)	0.158
No. of blastocysts (blastocyst rate)	9 (15.5%)	13 (25.0%)	0.214

*2PN represents the two-pronuclear zygote*.

*Fertilization rate is based on No. of 2PN/No. of MII*.

*Blastocyst rate is based on No. of blastocysts/No. of MII*.

### Distinct Gene Expression Profiles in GH Cultured Oocytes

**Figure 1:**
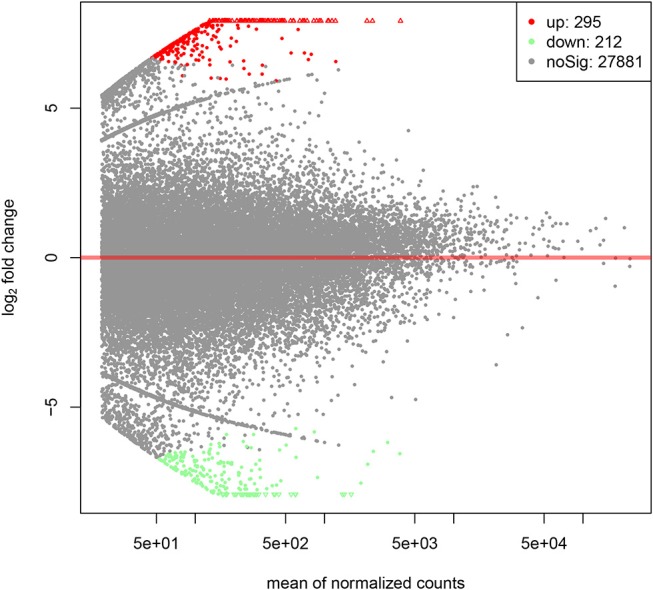
The MA plot of distinct gene expression profiles in GH cultured oocytes compared to the control group according to adjusted *P*-value < 0.05. X-axis represents the mean of normalized counts between the two groups; Y-axis represents the logarithm of fold change. From left to right in the X-axis, the expression level of the genes is from low to high. In the Y-axis, the more deviation it is from Y = 0, the larger the fold change is.

**Figure 2:**
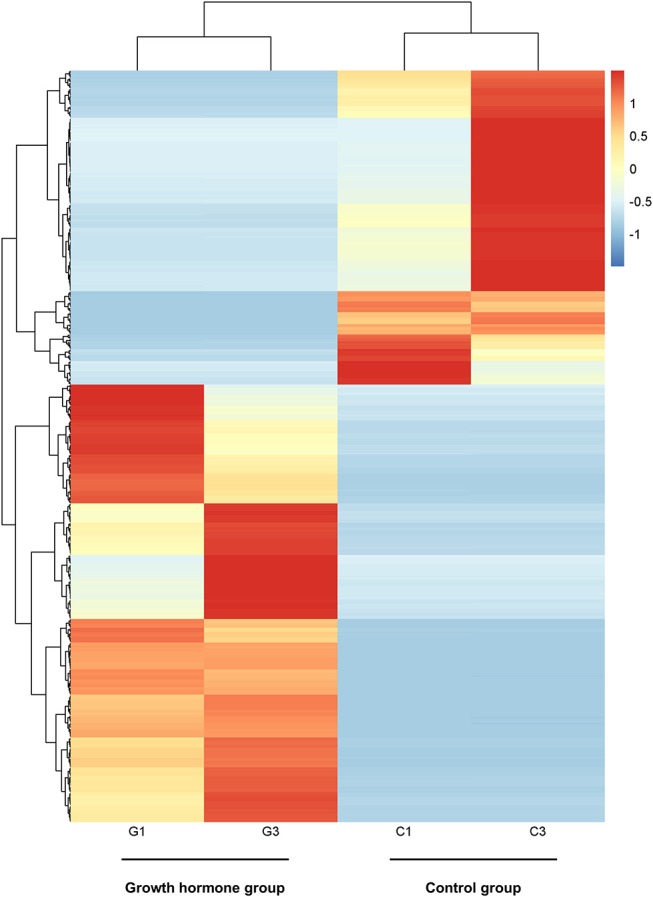
The heatmap of distinct gene expression profiles in GH cultured oocytes compared to the control group according to adjusted *P*-value < 0.05. Each row represents one gene expression levels among the four samples; Each column represents the expression levels of the genes in one sample; The black tree lines represent the results of layered clustering for the genes or samples, and the similar ones will be clustered. The different colors represent different expression levels and the illustration in the right represents the corresponding relationship between colors and the value of expression levels after standardization. G1and G3 represent samples from GH group; C1 and C3 represent samples from the control group.

By PCA (principal component analysis) for the three pairs of oocytes after RNA sequencing, we found one oocyte in the control group behaved differently compared to another two. Therefore, we only made use of the sequencing results from two pairs of oocytes. The MA plot showed that there were 295 up-regulated and 212 down-regulated transcripts which represented the corresponding genes according to adjusted *P*-value < 0.05 (Figure 1). In the heatmap, the control group samples and the experiment group samples were clustered respectively, which showed the consistency between the same kind samples. Dramatic changes of gene expression levels between the two groups were also revealed by the heatmap (Figure 2).

### Real-Time PCR for Validation

**Figure 3:**
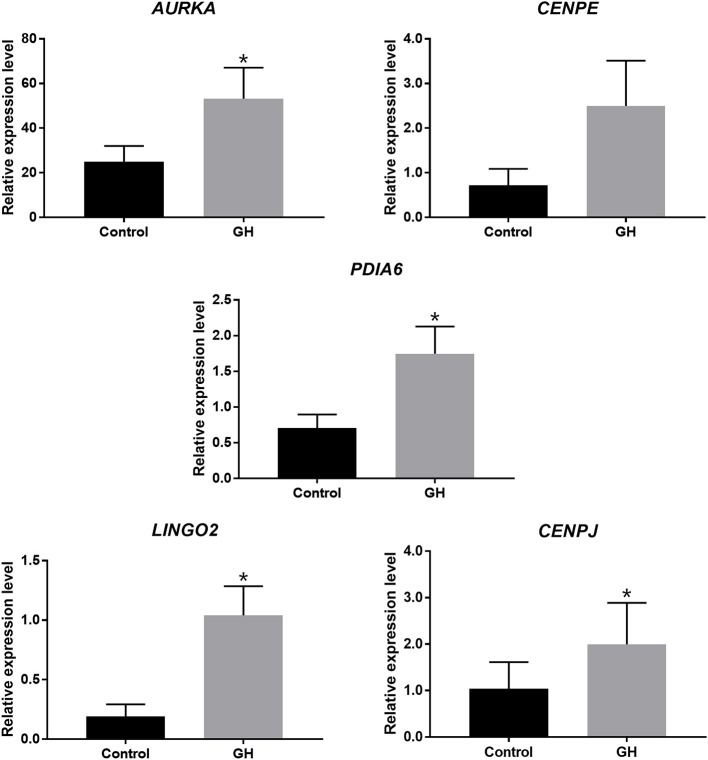
Real-time PCR for validation. ^*^P < 0.05.

In order to get the most significantly different genes between the two group, we filtrated the genes according to adjusted *P*-value < 0.001. The gene set was exhibited in Supplementary Table S2. Five genes were validated successfully in accordance with the sequencing results. They were as follows: *AURKA* (aurora kinase A), *CENPE* (centromere protein E), *PDIA6* (protein disulfide isomerase family A member 6), *LINGO2* (leucine rich repeat and Ig domain containing 2), and *CENPJ* (centromere protein J). Nine pairs of GV-stage oocytes from nine cases respectively were collected for *in vitro* culture with 200 ng/ml GH or not. After 24 h for IVM, the cDNA of these oocytes was obtained for validation, the results were displayed in Figure 3. The relative gene expression levels of *AURKA* (2.1-fold, *P* = 0.007), *PDIA6* (2.5-fold, *P* = 0.007), *LINGO2* (5.5-fold, *P* = 0.007), and *CENPJ* (1.9-fold, *P* = 0.039) were all significantly higher in the GH group compared to the control, and *CENPE* (3.5-fold, *P* = 0.098) tended to be increased by GH.

## Discussion

The clinical application of IVM is limited by its low success rate. In the present study, we found that GH could promote human oocyte maturation *in vitro*, and the concentration was optimized. We compared transcriptome profiles of human oocytes matured *in vitro* with GH or not by single-cell RNA-seq, which suggested that GH probably work by accelerating meiotic progression, balancing redox homeostasis of cellular environment, and promoting the oocyte developmental competence.

Building an IVM culture system with a higher success rate by adding effective ingredients has its clinical importance. In addition to the above-mentioned case report (26), it has only been reported that 1,000 ng/ml GH had no effect on IVM of human GV-stage oocytes, which were obtained during gynecologic surgery (31). Few studies have explored the optimal concentration of GH for human oocyte IVM. By increasing the concentration of GH gradually, we found that when it reached 200 ng/ml, the maturation rate reached the maximum. If the GH concentration was increased continually, instead, the maturation rate went down. Obviously, the 200 ng/ml was the optimal concentration. In this study, the maturation rate of IVM in the control group was 35%, which was close to the literature report of 38% (32). The maturation rate of 200 ng/ml GH group reached 70% which was double of the control group, and this indicated that GH could remarkably promote nucleus maturation of human oocytes. Furthermore, there have been studies identifying the presence of GH receptor on human oocytes (33, 34), and this may underlie the fact in this study that GH works in naked oocytes. The fertilization rate (from 60.3 to 73.1%) and blastocyst rate (from 15.5 to 25.0%) both had a tendency to be up-regulated by 200 ng/ml GH. No significant difference might be attributed to the sample size. This revealed that GH might have benefits on fertilization and early embryo development *in vitro*.

We further explored the roles of GH by single oocyte RNA-Seq. *AURKA* (aurora kinase A) in the GH group was 2.1-fold higher than the control with statistical significance. The intracellular localization of Aurora A is at the meiotic spindle poles and at a contractile ring/midbody during the first polar body extrusion, and a role in microtubule assembly and spindle organization is indicated (35–37). It has been reported that in *Xenopus* oocyte, the overexpression of Aurora A accelerated progesterone-induced GVBD (germinal vesicle breakdown) (38). In mouse oocyte, when Aurora A antibody was microinjected, the rate of GVBD was decreased (36). In porcine oocyte, Aurora A promoted the meiotic resumption (39). *CENPE* (centromere protein E) had a 3.5-fold increase in the GH group of this study. CENP-E is an essential meiotic kinetochore motor, and is required for meiotic progression; When mouse GV oocytes were injected with anti-CENP-E antibody, >95% of oocytes were arrested at MI even after 24 h, failing to extrude the first polar body (40). Therefore, in this study, GH might work through accelerating meiotic progression.

*PDIA6* (protein disulfide isomerase family A member 6) of GH group was significantly 2.5-fold higher than the control. *PDIA6* is a redox gene associated with redox homeostasis, and the expression of *PDIA6* was downregulated in *in vitro*-matured MII oocytes compared to *in vivo*-matured MII oocytes for both prepubertal and adult pigs (41). As we know, the *in vivo* maturation environment is more complete and more effective than *in vitro* for oocytes. In this study, *PDIA6* expression was up-regulated in the GH group, and this revealed that GH addition made the culture environment better for oocyte to balance the redox homeostasis.

*LINGO2* (leucine rich repeat and Ig domain containing 2) was remarkably increased by GH to 5.5-fold of the control. It has been reported that the expression level of the *Lingo2* gene increased gradually as the mouse embryo developed (42). This indicated that the more fully the embryo developed, the higher the expression level of *Lingo2* gene was. The expression level of *CENPJ* (centromere protein J) in the GH group was significantly up-regulated to 1.9-fold of the control. CENPJ is required for the biogenesis of centrioles, which organize centrosomes. For animal cells, centrosomes are the microtubule-organizing centers (43). What's more, centrosomes have an effect on cell polarity establishment, organelles positioning, and cell division organization (44). CENPJ is essential for centriole formation and defective centriole formation results in aberrant spindle positioning (45). While in the oocytes of most metazoan species, the centrioles and centrosomes are lacking (46). The zygotic centrosome will be restored at fertilization, and the functional zygote centrosome requires the blending of maternal centrosomal proteins and paternal reproducing element (47). The female cytosolic factors involved in the reformation of zygotic centrosomes are generated during oocyte meiosis in the preparation for fertilization, and the expression of centrosomal proteins in oocytes has already risen in meiosis II, just before fertilization (48). Thus, the expression of *CENPJ* in mature oocytes probably offers the preparation for centrosome restoring in zygote. In this study, by up-regulating *CENPJ* and *LINGO2*, GH might promote the developmental competence of the oocytes, and enable oocyte to reserve more useful materials in preparation for later fertilization and embryo development.

Taken together, this study identified that the optimal GH concentration 200 ng/ml could increase the success rate of human oocyte IVM. GH might play its roles by up-regulating *AURKA, PDIA6, LINGO2*, and *CENPJ*, which probably work through accelerating meiotic progression, balancing redox homeostasis of cellular environment, and promoting the oocyte developmental competence.

## Data Availability

The single-cell RNA-seq data in this study have been deposited in the GEO database, and the accession number is GSE133161.

## Ethics Statement

The study was approved by the Institutional Review Board of Reproductive Medicine, Shandong University. All human subjects gave written informed consent in accordance with the Declaration of Helsinki.

## Author Contributions

SZ designed the study. HZ and JM recruited the subjects. YL performed the experiments, analyzed the data, and wrote the paper. HL performed part of the experiments and analyzed the data. QY performed part of the experiments. H-BL and TH contributed to the reagents and materials, and assisted part of the experiments. SZ revised the manuscript and gave final approval of version to be published. All authors critically reviewed and approved the final manuscript.

### Conflict of Interest Statement

The authors declare that the research was conducted in the absence of any commercial or financial relationships that could be construed as a potential conflict of interest.
