# Supplementary material for: Growth Hormone Promotes in vitro Maturation of Human Oocytes
Source: Front Endocrinol (Lausanne). 2019 Jul 24;10:485. doi: 10.3389/fendo.2019.00485 (PMC6667636; doi:10.3389/fendo.2019.00485)
Supplement: Supplementary file 1 [file Table_1.docx]

**Supplemental Table S1**. Primer sequences for real-time PCR

| **Gene** | **Primer sequences (5'-3')** | **Product size(bp)** |
| --- | --- | --- |
| *GAPDH* | F: CATGAGAAGTATGACAACAGCCT  R: AGTCCTTCCACGATACCAAAGT | 113 |
| *AURKA* | F: GGAATATGCACCACTTGGAACA  R: TAAGACAGGGCATTTGCCAAT | 108 |
| *CENPE* | F: GATGACCTAGCAACTACACAGTC  R: AAAGCACCCAAACTCGAATCA | 185 |
| *PDIA6* | F: AGGAGGTCAGTATGGTGTTCAG  R: GAGGCGATCCTTCACGAGC | 151 |
| *LINGO2* | F: ACAATATGCCTGTGTATGCCTTT  R: ATGTGAGGTTGAGACCGTAGA | 117 |
| *CENPJ* | F: TCTCGGGCTGGGGTCATATTA  R: GAAAGGCTGTATGGGTTTCAGA | 226 |
