# Supplementary material for: Growth Hormone Promotes in vitro Maturation of Human Oocytes
Source: Front Endocrinol (Lausanne). 2019 Jul 24;10:485. doi: 10.3389/fendo.2019.00485 (PMC6667636; doi:10.3389/fendo.2019.00485)
Supplement: Supplementary file 2 [file Table_2.docx]

**Supplemental Table S2** Gene set according to adjusted *P*-value < 0.001

| Gene Name | Fold Change (GH group/control group) | *P*-value | Adjusted *P*-value |
| --- | --- | --- | --- |
| *AURKA* | 1810.17 | 0.0000000022 | 0.000033400 |
| *CENPE* | 1749.03 | 0.0000000025 | 0.000033400 |
| *TUBBP5* | 1644.68 | 0.0000000035 | 0.000033400 |
| *PDIA6* | 1120.01 | 0.0000000300 | 0.000190771 |
| *MED15* | 0.00091 | 0.0000000336 | 0.000190771 |
| *CKAP5* | 1038.23 | 0.0000000449 | 0.000197778 |
| *SLC7A7* | 1024.05 | 0.0000000488 | 0.000197778 |
| *KIF23* | 975.014 | 0.0000000622 | 0.000220596 |
| *RBBP8* | 916.953 | 0.0000000856 | 0.000258508 |
| *DDIAS* | 898.024 | 0.0000000984 | 0.000258508 |
| *CCAR1* | 876.753 | 0.0000001090 | 0.000258508 |
| *ZC2HC1B* | 0.00113 | 0.0000001080 | 0.000258508 |
| *GGNBP2* | 815.695 | 0.0000001560 | 0.000317335 |
| *EI24* | 799.737 | 0.0000001740 | 0.000329834 |
| *LINGO2* | 785.122 | 0.0000001910 | 0.000338062 |
| *USP10* | 772.286 | 0.0000002060 | 0.000344160 |
| *FAM13A* | 705.944 | 0.0000003410 | 0.000483778 |
| *SPECC1* | 705.883 | 0.0000003240 | 0.000483778 |
| *IWS1* | 700.857 | 0.0000003360 | 0.000483778 |
| *SIPA1L1* | 0.00147 | 0.0000003870 | 0.000522694 |
| *MED21* | 638.709 | 0.0000005340 | 0.000689425 |
| *MAP3K7CL* | 625.796 | 0.0000005850 | 0.000721960 |
| *CENPJ* | 597.934 | 0.0000007300 | 0.000840647 |
| *MELK* | 594.440 | 0.0000007510 | 0.000840647 |
| *SMC6* | 586.754 | 0.0000007980 | 0.000840647 |
| *FDFT1* | 0.00171 | 0.0000008000 | 0.000840647 |
| *ARID3B* | 0.00177 | 0.0000009560 | 0.000969280 |
